# Pancreatic stone protein: a new predictor of outcome in patients with peritonitis

**DOI:** 10.1186/cc11749

**Published:** 2012-11-14

**Authors:** R Gukasjan, D Raptis, HU Schulz, W Halangk, R Graf

**Affiliations:** 1 Otto-von-Guericke University, Magdeburg, Germany; 2 University Hospital Zurich, Switzerland

## Background

Infection and sepsis are serious postoperative complications, which have to be recognized and treated by appropriate therapeutic options in the ICU. Pancreatic stone protein (PSP/*reg*) is synthesized and secreted by the pancreas, the stomach and the small intestine. In this study we evaluated whether PSP/*reg *predicts sepsis-related postoperative complications and death in patients with peritonitis.

## Methods

A prospective cohort study of postoperative patients admitted to the ICU in an adult surgical teaching hospital in Germany. Ninety-one consecutive postoperative patients with proven diagnosis of secondary peritonitis admitted to the ICU were included in the study. Blood samples were taken within 3 hours from admission to the ICU for analysis of PSP/*reg*, white blood cell counts (WCC), C-reactive protein (CRP), IL-6, and procalcitonin (PCT). The Mannheim Peritonitis Index and APACHE II clinical scores were also determined. Univariate and multivariate analyses were performed to determine the diagnostic accuracy and independent predictors of death in the ICU.

## Results

Univariate analysis demonstrated that PSP/*reg *has the highest diagnostic accuracy for complications such as organ failure and is the best predictor for death in the ICU. PSP/*reg *had the highest overall efficacy in predicting death with an odds ratio (OR) of 4.0 versus PCT (OR 3.2), IL-6 (OR 2.8), CRP (OR 1.3), and WCC (OR 1.4). By multivariate analysis, PSP/*reg *was the only independent predictor of death.

## Conclusion

Patients with sepsis-related complications showed elevated serum-PSP/*reg *levels. PSP/*reg *demonstrated a high diagnostic accuracy to discriminate the severity of peritonitis and to predict death in the ICU. This test could be used in clinical diagnosis and therapeutic decision-making in the ICU.

